# Analysis of the use of rosemary for cortisol levels and sleep quality among nursing professionals

**DOI:** 10.1590/1980-220X-REEUSP-2025-0050en

**Published:** 2025-09-29

**Authors:** Nathália Fortes Schlotfeldt, Rosângela Marion da Silva, Etiane de Oliveira Freitas, Fernanda Moura D’Almeida Miranda, Carolina Renz Pretto, Flávia Camef Dorneles Lenz

**Affiliations:** 1 Universidade Federal de Santa Maria, Programa de Pós-Graduação em Enfermagem, Santa Maria, RS, Brazil.; 2 Universidade Federal do Paraná, Curitiba, PR, Brazil.; 3 Secretaria Municipal de Saúde de Ijuí, Serviço de Atendimento Móvel de Urgência, Ijuí, RS, Brazil.

**Keywords:** Emergency Nursing, Sleep, Rosmarinus, Hydrocortisone

## Abstract

**Objective:**

To analyze the effectiveness of 1 g of dry rosemary extract on sleep quality and hair cortisol concentration in emergency nursing professionals.

**Method:**

Intervention, quasi-experimental, before-and-after study, carried out between September and November 2022 with nursing professionals from a hospital emergency room and an Emergency Room in Rio Grande do Sul. A sociodemographic/occupational questionnaire, Pittsburgh Sleep Quality Index, and hair samples were used. The intervention involved taking 500 mg capsules of dry rosemary extract twice a day for 56 days. Descriptive and analytical analysis, with association and correlation tests (p < 0.05) were used.

**Results:**

Thirty-five workers participated. Poor sleep quality and high levels of hair cortisol concentration predominated. The intervention significantly improved sleep (p = 0.039), without reducing cortisol levels.

**Conclusion:**

The rosemary intervention improved sleep quality, but was not sufficient to reduce hair cortisol concentrations, with other strategies that contribute to the worker’s health being required.

## INTRODUCTION

Sleep is important for homeostasis, when the waking state is interrupted, leading to a decrease in metabolic activities, muscle relaxation, and a reduction in sensory activities^(1)^. Sleep interferes with cognitive functions, memory consolidation and information storage, and is essential for the human body balance^(2)^.

Sleep disorders affect the ability to sleep and have been linked to changes in cortisol levels, known as the stress hormone, which impacts physical and mental health. Cortisol follows a natural circadian cycle, peaking in the morning to prepare the body for daily activities and decreasing throughout the day to facilitate sleep. However, sleep problems, such as insomnia or poor quality sleep, can alter this rhythm by maintaining elevated cortisol levels at night, which contributes to chronic stress and fatigue^(3)^.

This situation, combined with the demands of the job and shift work, implies the worker falling ill. Professionals who work in shifts, such as nurses working in emergency services, face stressful situations, time pressure, and physical demands^(4)^. Night shift work, in particular, involves circadian desynchronization as workers have to stay awake during the period in which their bodies are physiologically prepared for rest, which highlights the importance of interventions that can contribute to the workers’ quality of sleep and health.

To improve the quality of sleep, the use of medicinal plants, such as *Rosmarinus officinalis L.* (rosemary), is mentioned as an alternative. This plant is widely used as a natural food preservative, presenting pharmacological actions that include antibacterial, antidiabetic, anti-inflammatory, antitumor, and antioxidant properties^(5)^. Despite limited intervention research with rosemary, evidence points to benefits such as reduced anxiety, improved depression sleep quality^(6)^, decreased daytime sleepiness^(7)^. These results suggest that rosemary is a therapeutic possibility for improving sleep; however, there is no evidence linking its use to improving stress.

Chronic stress can be assessed through hair cortisol measurement. Hair samples have the advantage of detecting cortisol over several months and are a non-invasive procedure, besides being easy to store and resistant to decomposition, unlike bodily fluids^(8)^.

Thus, considering the limited literature available on the effects of rosemary on human health, the recent reduced number of intervention studies on hair cortisol, and that nursing professionals are susceptible to stress, the importance of analyzing the effectiveness of 1 g of dry rosemary extract on the quality of sleep and hair cortisol concentration of nursing professionals in emergency services is identified, which is the objective of this research.

## METHOD

### Study Design

This is a quasi-experimental study, with a single group, pre and post-intervention.

### Population and Local

It was carried out in a municipality in the northwest region of the State of Rio Grande do Sul, in a 24-hour Emergency Care Unit (*UPA*), a public institution that provides emergency and urgent care at an intermediate level to a population of 83,764 inhabitants and has 43 nursing professionals, and in a hospital Emergency Room (*PSH*), which is part of a philanthropic institution that is a reference in health care to all the region covered by the 17th Regional Health Coordination, which covers a population of approximately 231,229 people and has 57 nursing professionals in the sector.

### Selection Criteria

Nursing technicians and assistants and nurses who were working in the emergency services of the selected institutions for at least one month, under any form of contract, were included. The service time delimitation took into account the Pittsburgh Sleep Quality Index, which assesses the quality of sleep over the previous month. Regarding exclusion criteria, they were: professionals who presented hypersensitivity to rosemary; pregnant women; lactating women; epileptics; those undergoing treatment for dyslipidemia or diabetes mellitus; professionals with biliary or hepatic dysfunction, with prostate diseases or gastroenteritis, due to the lack of studies ensuring the safety of use under these conditions; professionals with hair strands < 2 cm in the posterior vortex region of the head or who were using topical medication on the scalp during the data collection period, to prevent interference in cortisol concentration; or those away from work for any reason during the data collection period.

### Sample Definition

The sampling was non-random and the minimum sample was established through sample calculation, with the application of an expression for paired proportions^(9)^, in which a proportion of 50% of concordant and 10% of discordant, a certainty (test power) of 80%, and a 5% significance level were arbitrated. These parameters resulted in a minimum sample size of 19 participants, taking into account that each one would be their own peer in the evaluation of the outcomes before and after the intervention. The study population comprised 100 nursing professionals and all were invited to participate in the research.

### Data Collection

The research comprised two stages of data collection (before and after the intervention) and an intervention period. The collection took place from September to November 2022, individually and in person, at the participants’ workplace, with prior scheduling and subject to availability. Two people concomitantly assisted in the collection, a nursing undergraduate student with an undergraduate research scholarship, who received training and qualification for data collection, and a nurse, with experience in quantitative data collection, a nursing doctoral student.

### Data Collection Instrument

Data collection instruments comprised self-administered questionnaires with sociodemographic variables and the Pittsburgh Sleep Quality Index (PSQI-BR). Hair samples were also collected for hair cortisol analysis.

The sociodemographic and clinical questionnaire was used to characterize the participants and included the investigation of the variables: age (in years), sex assigned at birth (male, female), marital status (with partner/without partner), children (yes, no), workplace (*UPA, PSH*), position (nurse, nursing technician, nursing assistant), working hours (≤ 44 hours/week, > 44 h/week), number of employment contracts (one, more than one), smoking (yes, no), sleeping medication (yes, no), and use of herbal medicines (yes, no).

The PSQI-BR questionnaire, a version translated and validated for Brazilian Portuguese, was used to measure sleep quality^(10)^. This questionnaire has 19 questions organized into seven components: subjective sleep quality (C1), sleep latency (C2), sleep duration (C3), habitual sleep efficiency (C4), sleep disturbances (C5), use of sleeping medication (C6) and daytime dysfunction (C7), and five directed to the spouse or roommate, regarding sleeping habits during the past month. For this study, issues related to spouses were not investigated. The sum of the scores for each item on the instrument results in an overall score ranging from 0 to 21. A total score greater than 5 indicates that the individual is experiencing major dysfunction in at least two components, or moderate dysfunction in at least three components^(10)^. For this study, we chose to categorize the variable sleep quality as “good” (≤ 5) and “bad” (> 5 points), as previously identified in a study^(11)^.

Cortisol levels were measured using hair strands ≥ 2 cm in length (measured with a metal ruler with a 10 cm/0.1 mm cut), removed as close as possible to the root, at the back vertex of the head, and stored individually in containers, without freezing and protected from light, as recommended^(12)^.

The samples underwent cortisol extraction and quantification using the Enzyme-Linked Immunosorbent Assay – ELISA method. Following collection, the strands were washed with isopropanol, sectioned, and incubated in methanol (MeOH) at 50°C for 16 hours to extract steroids. After drying, the residue was reconstituted in 250 µL of phosphate-buffered saline (PBS) and the sample was vortexed for cortisol quantification, according to adapted protocol^(13)^.

Considering that hair grows at a relatively constant rate of approximately one centimeter per month, it is said that the three centimeters of hair closest to the scalp reflect the cortisol levels to which the individual has been exposed over the past three months^(14)^. For healthy individuals with low levels of stress, the median hair cortisol concentration is 55 picograms of cortisol/milligrams of hair (pg/mg) (40–128 pg/mg) and 250 pg/mg of hair (182–520 pg/mg) in stressed individuals^(14)^. In this study, the mean hair cortisol level before the intervention was 600.30 pg/mg (SD = 6.03); therefore, a cutoff point of less than or equal to 600 pg/mg and greater than 600 pg/mg was used for the analyses.

The individual approach included information about the research and criteria for participation. For those interested, the eligibility criteria were applied and the delivery of the Free and Informed Consent Form was delivered, with its reading and consent to participate being required by means of a signature, in two copies, one for the participant and the other for the research team. Later, hair strands were collected and kept in a hermetic container, protected from sunlight. The questionnaires were delivered, with a date scheduled for their return, and the rosemary capsules for the intervention were made available. Participants authorized the sending of messages via electronic messaging applications.

The intervention consisted of providing each participant with a bottle containing 60 capsules of 500 mg of dry extract of *Rosmarinus officinalis L.* (rosemary) to be taken with water, two times a day (one capsule in the morning, if possible, 30 minutes after breakfast, and another 12 hours after the first), totaling 1 g/day, for a period of 56 days. The dry extract SD 0.03% of *Rosmarinus officinalis L*. originates from the leaves of the plant, with the appearance of a fine hygroscopic powder without the presence of foreign materials, greenish-beige in color, with a characteristic odor and flavor, with total ash of 63 in values lower than 5.0%, density between 0.300 and 0.600, pH between 4.0 and 8.0, partially water-soluble, with humidity lower than 10.0%, active flavonoid content greater than 0.03, aerobic bacteria count lower than 10,000, fungi or yeast count lower than 1,000, and enterobacteria count lower than 1,000.

For greater safety and control regarding adherence to the proposed treatment, each participant was provided with a mini diary to record the time of self-administered doses, forgotten doses, and those delayed; and a leaflet with instructions on how to continue taking routine medications, including teas and herbal medicines, and the doses and times of the herbal medicine self-administration, what to do if the dose was forgotten, care for conservation, and how to contact researchers in case of doubts or complications. The participant was advised that, in cases when the daily dose was forgotten, the forgotten capsule should be returned to the researcher at the end of the intervention period (at the end of 56 days), as a way of controlling the doses ingested.

During the intervention period, messages were sent every five days via the app to encourage the use of herbal medicine and minimize doubts. The participant was instructed to communicate the research team immediately of any complications arising from the use of the herbal medicine, and decide whether to remain in the study or withdraw. At the end of the 56 days, a new date was scheduled for data and hair samples collection.

### Data Analysis and Treatment

The collected data formed a bank in the software Microsoft Excel® through independent double typing, and were analyzed with the help of the software SPSS, version 21.0. They are stored on the research coordinator’s personal computer and may be made available upon consultation. Quantitative variables were expressed as mean and standard deviation in the presence of normal distribution, or median and interquartile range in the presence of asymmetric distribution. Categorical variables were presented using absolute and relative values; quantitative variables, by the mean and standard deviation. To investigate an association between categorical variables, Pearson’s Chi square test or Fisher’s exact test was used. For the analysis of paired samples, the McNemar Test was used. Spearman’s correlation analysis was performed to assess the relationships between the study variables. Confidence interval of 95% and p < 0.05 were considered.

### Ethical Aspects

The study is registered in the Brazilian Clinical Trials Registry – Rebec (no. RBR 88hrnnw). It was approved by the Research Ethics Committee with no. 5,197,916, meeting the ethical precepts of research as per Law No. 14,874/2024, which provides for research involving human beings. Participation was voluntary, and consent for the research occurred after the Free and Informed Consent Form was read and signed.

## RESULTS

Forty-one participants refused to participate. When the eligibility criteria were applied, 11 were excluded (three were breastfeeding, one was pregnant, one was undergoing treatment to become pregnant, two had hair of length < 2 cm, one was undergoing scalp treatment, one had a digestive disease, and two were undergoing treatment for diabetes mellitus). After the intervention began, 12 professionals stopped using the herbal medicine and did not participate in the final phase of data collection. Furthermore, one participant was excluded due to incomplete completion of the sleep questionnaire, which made the analysis unfeasible.

Thirty-five nursing workers with a mean age of 39.67 years (± 7.58) participated. The women participating accounted for 85.7% (n = 30) of the total, 80% (n = 28) had children, 74.3% (n = 26) had a partner. The majority worked at the *UPA* (77.1%, n = 27) and were nursing technicians or assistants (77.1%, n = 27), with a workload ≤ 44 hours/week, and had one job. Regarding the use of medications, there was a prevalence of professionals who did not use medications to sleep (85.7%, n = 30) and who did not use herbal medicine (97.1%, n = 34).

It was found that, **before the intervention**, poor sleep quality predominated in those with a partner (68.2%), with children (72.7%), with one job, and a workload of 44 hours/week (77.3%) or more. **After the intervention**, poor sleep quality predominated in those with a partner and children (66.7%), with one job, and a workload of 44 hours/week (86.7%) or less.

Regarding cortisol levels, **before the intervention** it was observed that 86.7% (n = 13) of the female participants had cortisol values greater than 600 pg/mg, as did those who had a partner (86.7%, n = 13) and who had children (93.3%, n = 14). Most professionals who did not use sleeping medication (90%, n = 18) and those who did not use herbal medicine (100%, n = 20) presented cortisol levels < 600 pg/mg.

It was found that, **after the intervention**, there was no significant association between cortisol levels and sociodemographic and occupational variables. That is, in this sample, number of jobs and workload did not affect cortisol levels after the intervention.

On Table 1, there are data related to sleep quality and cortisol levels before and after the intervention with rosemary.

**Table 1:** Relationship between sleep quality before and after the intervention in emergency service nursing professionals – Ijuí, RS, Brazil, 2023. (n = 35).

	Sleep quality		p
	Good	Poor	
Before	13 (37.1)	22 (62.9)	0.039*
After	20 (57.1)	15 (42.9)	
	**Hair cortisol concentration**		**p**
	**<600 pg/mg**	**≥600 pg/mg**	
Before	20 (57.1)	15 (42.9)	<0.001*
After	2 (5.7)	33 (94.3)	

Source: Authors, 2023.

*McNemar test.

There was a significant difference between the quality of sleep classified as good, when comparing the period before and after the intervention (p = 0.039). An increased proportion of professionals with cortisol levels greater than or equal to 600 pg/mg was also observed, when the periods before and after the intervention (p < 0.001) were compared.

It was found that those who used sleeping medication had the worst cortisol levels (p = 0.017). No association between the use of medication and herbal medicine and sleep quality was observed.

Table 2 presents the correlation matrix, which shows that sleep quality before the intervention was positively related to sleep quality after the intervention; cortisol levels before the intervention were positively related to the number of jobs; and working hours were positively related to cortisol levels after the intervention.

**Table 2:** Correlation matrix between study variables of nursing professionals in emergency services – Ijuí, RS, Brazil, 2023. (n = 35).

	Age	Employment relationship	WL	SQ before intervention	SQ after intervention	Cortisol before intervention
Sleep quality before	.054	−.057	−.044			
	.758	.746	.803			
Sleep quality after	−.094	−.229	−.045	.673**		
	.590	.187	.799	**.000**		
Cortisol before intervention	.205	.364*	.094	.058	−.277	
	.238	**.031**	.593	.739	.107	
Cortisol after the intervention	.035	.321	.360*	.000	−.190	.209
	.843	.060	**.034**	.998	.275	.228

Note: WL – Workload; SQ – Sleep Quality.

*Significant correlation at p ≤ 0.05.

**Significant correlation at p ≤ 0.01 level.

## DISCUSSION

The results showed that the intervention with 1 g of dry extract of *Rosmarinus officinalis L.* (rosemary) improved the sleep quality of emergency service nursing professionals, as a significant difference was identified in sleep quality before and after the intervention; however, it was not enough to reduce hair cortisol levels in the group investigated.

In this study, the female sex assigned at birth predominated, a result similar to a national study carried out in emergency units^(15)^. A study found that women were about twice as likely to have poorer sleep quality than men, which may be related to psychological conditions, since they present symptoms of anxiety and depression more frequently than men and hormonal changes, present during menstrual cycles, pregnancy and menopause^(1)^. Moreover, there is a risk of stress given the nature of the working conditions^(16)^, influencing sleep patterns.

Poor sleep, resulting from sleep deprivation, has effects on mood that are comparable to the effects on cognitive functioning. This finding is relevant for emergency care workers, due to critical events involving care. In this study, a predominance of poor sleep quality was identified before the intervention (62.9%, n = 22), datum similar to studies carried out with nursing that used the same assessment instrument^(11)^.

Poor sleep quality occurs when an individual cannot fall asleep, wakes up several times during the night, and does not feel rested upon waking up, and this condition is closely linked to the worsening of cardiovascular disease^(17)^. Evidence from a meta-analysis led to the conclusion that nurses are the professionals most susceptible to poor sleep quality^(18)^.

Other variables can influence sleep quality, such as having children, working hours, and marital status. The results showed that before and after the intervention there was poor sleep quality in those who had children, which partially agrees with the results of a study that showed that having small children significantly influences the quality of parents’ sleep^(19)^. The lack of questions about the children’s ages prevented a more in-depth analysis of the data.

There was a prevalence of poor sleep quality in those with a partner, a finding that differs from research carried out with Australian workers, which found that married individuals had better sleep quality^(19)^.

The use of rosemary improved the quality of sleep. Before the intervention, 37.1% of participants had good sleep quality; after the intervention, this percentage rose to 57.1%. Similarly, 62.9% of participants had poor sleep quality; after the intervention, this percentage was 42.9%. This means that the intervention influenced the improvement of sleep quality in this sample. To have good quality sleep is important for maintaining health, as some toxin clearance mechanisms occur during this period and given its relevance to immunological, cardiovascular, reproductive, endocrine functions and pain control^(2)^.

The results of rosemary’s effect on sleep quality are groundbreaking in Brazilian publications, and are similar to the results of Japanese research that observed a reduction in daytime sleepiness with the intake of 1 g/day of a test food containing dry extract of Rosmarinus officinalis L. for 4 weeks^(7)^. A study carried out with rosemary in its essential oil form was able to improve the quality of sleep in anxious individuals with difficulty relaxing, helping to regulate the sleep-wake cycle^(6)^.

The benefits of rosemary have been evidenced in the literature, such as the reduction of symptoms of common mental disorders, such as irritation, anxiety, difficulty sleeping and difficulty concentrating, without adverse effects^(20)^ and as an aid to diet, with a reduction in body mass index and waist- hip ratio^(21)^.

In relation to **cortisol**, before and after the intervention there was a prevalence in females, with values > 600 pg/mg, which characterizes high rates, although not statistically significant. Research conducted with nursing professionals identified high levels of hair cortisol, which suggests the presence of stress, but without statistical significance^(22)^. Another study found an association between high levels of hair cortisol and perceived stress^(4)^. A study highlights that interventions focused on worker well-being can mitigate some of these negative effects by reducing stress and anxiety among nurses^(23)^.

A significant increase in hair cortisol levels could be observed in the sample studied after the intervention, which suggests chronic stress in this professional group. This may be related to inconsistencies in the work process in the emergency department, where dealing with the constant state of readiness and human suffering can have negative impacts on the nursing professionals’ health and well-being. Individuals with elevated cortisol levels showed significant increases in total cholesterol, LDL, and triglyceride levels^(24)^, perceived stress^(4)^, and greater severity of insomnia, depression and anxiety^(25)^.

Data also shows a correlation between having more than one job and worse cortisol levels, as well as higher cortisol levels in those who worked longer hours, showing that the higher the number of jobs and the workload, the higher the stress level. Emergency nurses are highly susceptible to burnout due to continued exposure to traumatic events, varying work schedules, violence directed at staff, and, more recently, due to stressors arising from the COVID-19 pandemic^(26)^. Stress among emergency room workers is linked to increased hair cortisol concentration and physiological reactions such as back pain, fatigue, exhaustion, neck stiffness, and heartburn^(22)^.

There was a significant association between cortisol and the use of sleeping medication after the intervention, that is, workers who used sleeping medication had higher cortisol levels. A study carried out with nursing professionals during the COVID-19 pandemic found a higher prevalence of poor sleep reported by 85.7% of the sample, and the use of sleeping pills by 25.9% of them^(27)^, suggesting that these professionals seek medicinal solutions to improve sleep quality.

When analyzing the relationship between hair cortisol and sleep quality, no significant association was identified before and after the intervention. Despite this, researchers emphasize the importance of measuring hair cortisol in nursing professionals, obtained from hair samples, as it is a reliable biomarker for measuring stress^(16)^.

Poor sleep quality is linked to negative health impacts, such as physical illness^(12)^, appetite disorders, feeling of indigestion, flatulence, weight gain, irritability, headache, feeling of low self-esteem, mood lability^(2)^. Individuals who are sleep deprived have their cortisol levels altered as a response to stress, as sleep deprivation and disruption affect the circadian rhythm and immune system^(28)^.

When interpreting the results of this study, it is important to highlight limitations such as the small sample size and the use of self-administered questionnaires, whose data may be biased. Another point to be considered is the 2 cm hair sample required for cortisol analysis, which limited the number of male participants.

The lack of effect of rosemary on chronic stress can be explained by the short period of data collection. Researchers suggest that hair cortisol reflects hormonal variations over longer timescales, such as months to a year, and is not sensitive to rapid changes associated with acute stress^(29)^. Therefore, further studies are required, with longitudinal designs, that consider a longer follow-up period and with larger samples that may have the potential to broaden the understanding of the effects of rosemary on cortisol with a view to developing more effective preventive and treatment interventions. Future research may uncover the lasting effects of rosemary on human health.

The study contributes with data regarding nursing work in emergency services and can contribute to the planning of actions that promote worker health through integrative health practices. Moreover, it provides data on the assessment of hair cortisol, expanding the possibilities for investigating this biomarker. The results provide evidence for the possible effect of rosemary on sleep quality, with possibilities for developing new preventive and treatment strategies.

## CONCLUSION

The intervention with 1 g of dry rosemary extract significantly improved sleep quality and was associated with increased cortisol levels in nursing professionals in emergency services. The limited evidence on this topic requires further investigation, and the data suggest the development of strategies that help minimize stress and improve the quality of sleep for these professionals.

## DATA AVAILABILITY

The entire dataset supporting the findings of this study is available upon request to corresponding author Nathália Fortes Schlotfeldt. The dataset is not publicly available because it has not yet reached the minimum two-year validity period required for its availability.
