# Supplementary material for: Analysis of the use of rosemary for cortisol levels and sleep quality among nursing professionals
Source: Rev Esc Enferm USP. 2025 Sep 29;59:e20250050. doi: 10.1590/1980-220X-REEUSP-2025-0050en (PMC12478303; doi:10.1590/1980-220X-REEUSP-2025-0050en)
Supplement: Translation

# Translation: Análise do uso do alecrim para o nível de cortisol e qualidade do sono entre profissionais de enfermagem

**DOI:** 10.1590/1980-220X-REEUSP-2025-0050pt

**Authors:** Nathália Fortes Schlotfeldt, Rosângela Marion da Silva, Etiane de Oliveira Freitas, Fernanda Moura D’Almeida Miranda, Carolina Renz Pretto, Flávia Camef Dorneles Lenz

**Affiliations:** 1 Universidade Federal de Santa Maria, Programa de Pós-Graduação em Enfermagem, Santa Maria, RS, Brasil.; 2 Universidade Federal do Paraná, Curitiba, PR, Brasil.; 3 Secretaria Municipal de Saúde de Ijuí, Serviço de Atendimento Móvel de Urgência, Ijuí, RS, Brasil.

**Keywords:** Enfermagem em Emergência, Sono, Rosmarinus, Hidrocortisona

## Abstract

**Objetivo:**

Analisar a efetividade de 1 g de extrato seco do alecrim na qualidade do sono e cortisol capilar de profissionais de enfermagem de urgência/emergência.

**Método:**

Estudo de intervenção, quase experimental, do tipo antes e depois, realizado entre setembro e novembro de 2022 com profissionais de enfermagem de um pronto-socorro hospitalar e de um Pronto Atendimento do Rio Grande do Sul. Utilizaram-se questionário sociodemográfico/ocupacional, Índice de Qualidade do Sono de Pittsburgh e amostras de cabelo. A intervenção compreendeu a ingestão de cápsulas de extrato seco de alecrim de 500 mg, duas vezes ao dia, por 56 dias. Análise descritiva e analítica, com testes de associação e correlação (p < 0,05).

**Resultados:**

Participaram 35 profissionais. Predominaram a má qualidade do sono e níveis elevados de cortisol capilar. A intervenção melhorou significativamente o sono (p = 0,039), sem redução nos níveis de cortisol.

**Conclusão:**

A intervenção com o alecrim melhorou a qualidade do sono, mas não foi suficiente para reduzir as concentrações de cortisol capilar, fazendo-se necessárias outras estratégias que colaborem com a saúde do trabalhador.

## INTRODUÇÃO

O sono é importante para a homeostase, momento em que o estado de vigília é interrompido, levando a uma diminuição das atividades metabólicas, relaxamento muscular e redução das atividades sensoriais^(1)^. O sono interfere nas funções cognitivas, consolidação da memória e no armazenamento de informações, e é essencial para o equilíbrio do organismo humano^(2)^.

Os distúrbios do sono afetam a capacidade de dormir e têm sido associados a alterações nos níveis de cortisol, conhecido como o hormônio do estresse, com impacto na saúde física e mental. O cortisol segue um ciclo circadiano natural, atinge seu pico pela manhã, para preparar o corpo para as atividades diárias, e diminui ao longo do dia para facilitar o sono. No entanto, problemas no sono, como insônia ou sono de baixa qualidade, podem alterar esse ritmo ao manterem níveis elevados de cortisol à noite, o que contribui para o estresse crônico e fadiga^(3)^.

Essa situação somada às exigências do trabalho e a realização do trabalho em turnos sugerem adoecimento do trabalhador. Profissionais que trabalham em turnos, como os da enfermagem que atuam em serviços de urgência e emergência, enfrentam situações de estresse, pressão de tempo e as exigências físicas^(4)^. O trabalho no turno noturno, em especial, implica em dessincronização circadiana pelo motivo de o trabalhador precisar se manter acordado no período que o organismo, fisiologicamente, está preparado para o descanso, o que alerta para a importância de intervenções que possam contribuir para a qualidade do sono e saúde do trabalhador.

Para melhorar a qualidade do sono, cita-se como alternativa o uso de plantas medicinais, como o *Rosmarinus officinalis L.* (alecrim). Essa planta é amplamente utilizada como conservante natural de alimentos, apresentando ações farmacológicas que incluem antibacterianas, antidiabéticas, anti-inflamatórias, antitumorais e antioxidantes^(5)^. Apesar de limitadas pesquisas de intervenção com o alecrim, evidências apontam benefícios, como a redução de ansiedade, melhora da depressão e da qualidade do sono^(6)^, diminuição da sonolência diurna^(7)^. Estes resultados sugerem que o alecrim é uma possibilidade terapêutica para melhorar o sono; no entanto, não há evidências que relacionem seu uso para melhorar o estresse.

O estresse crônico pode ser avaliado pela dosagem do cortisol capilar. As amostras de cabelo têm como vantagens a detecção do cortisol ao longo de vários meses e abrangem um procedimento não invasivo e serem de fácil armazenamento e resistência à decomposição, ao contrário dos fluidos corporais^(8)^.

Assim, considerando a restrita literatura disponível sobre os efeitos do alecrim na saúde humana, o reduzido quantitativo recente de estudos de intervenção sobre o cortisol capilar, e que profissionais de enfermagem são suscetíveis ao estresse, identifica-se a importância de analisar a efetividade de 1 g de extrato seco do alecrim na qualidade do sono e na concentração de cortisol capilar de profissionais de enfermagem de serviços de urgência/emergência, objetivo desta pesquisa.

## MÉTODO

### Tipo de Estudo

Trata-se de um estudo quase experimental, com um único grupo, pré e pós-intervenção.

### Local e População

Foi realizado em um município da região noroeste do Estado do Rio Grande do Sul, em uma Unidade de Pronto Atendimento (UPA) 24h, instituição pública que presta assistência de urgência e emergência em nível intermediário a uma população de 83.764 habitantes e conta com atuação de 43 profissionais de enfermagem; e um pronto-socorro hospitalar (PSH), que integra uma instituição filantrópica referência de atendimento em saúde a toda a região coberta pela 17ª Coordenadoria Regional de Saúde, que abrange uma população de aproximadamente 231.229 pessoas e que conta com 57 profissionais de enfermagem no setor.

### Critérios de Seleção

Foram incluídos os técnicos e auxiliares de enfermagem e os enfermeiros que estavam atuando nos serviços de emergência das instituições selecionadas havia pelo menos um mês, sob qualquer forma de contratação. A delimitação do tempo de serviço levou em conta o Índice de Qualidade do Sono de Pittsburgh, que avalia a qualidade do sono referente ao mês anterior. Quanto aos critérios de exclusão, foram desconsiderados: profissionais que apresentavam hipersensibilidade ao alecrim; gestantes, lactantes, epilépticos, aqueles em tratamento para dislipidemia ou diabetes mellitus, profissionais com disfunção biliar ou hepática, com doenças prostáticas ou gastroenterite, devido à falta de estudos que garantam a segurança do uso nestas condições; profissionais com fios de cabelo < 2 cm na região do vórtice posterior da cabeça ou que estavam utilizando medicação tópica no couro cabeludo durante o período de coleta de dados, para prevenir interferência na concentração de cortisol; ou afastados do trabalho por qualquer razão durante o período de coleta de dados.

### Definição da Amostra

A amostragem foi não aleatória e a amostra mínima foi estabelecida por meio de cálculo amostral, com aplicação de uma expressão para proporções pareadas^(9)^, na qual se arbitraram uma proporção de 50% de concordantes, 10% de discordantes, uma certeza (poder do teste) de 80% e 5% de nível de significância. Esses parâmetros resultaram no quantitativo amostral mínimo de 19 participantes, levando em conta que cada um seria seu próprio par na avaliação dos desfechos antes e depois da intervenção. A população de estudo compreendeu 100 profissionais de enfermagem e todos foram convidados a participar da pesquisa.

### Coleta de Dados

A realização da pesquisa compreendeu duas etapas de coleta de dados (antes e depois da intervenção) e um período de intervenção. A coleta ocorreu de setembro a novembro de 2022, de forma individual e presencial, no local de trabalho dos participantes, com agendamento prévio e conforme disponibilidade. Auxiliaram concomitantemente na coleta duas pessoas, uma graduanda em enfermagem, bolsista de iniciação científica, que recebeu capacitação e treinamento para a coleta de dados, e uma enfermeira, com experiência em coleta de dados quantitativos, doutoranda em enfermagem.

### Instrumento de Coleta de Dados

Os instrumentos de coleta de dados compreenderam questionários autoaplicáveis, com variáveis sociodemográficas, e o Índice de Qualidade do Sono de Pittsburgh (PSQI-BR). Também foram coletadas amostras de cabelo para análise de cortisol capilar.

O questionário sociodemográfico e clínico foi utilizado para a caracterização dos participantes e incluiu a investigação das variáveis: idade (em anos), sexo atribuído no nascimento (masculino, feminino), situação conjugal (com companheiro/sem companheiro), filhos (sim, não), local de trabalho (UPA, PSH), cargo (enfermeiro, técnico de enfermagem, auxiliar de enfermagem), carga horária (≤ 44 h/semanais, > 44 h/semanais), número de vínculos de trabalho (um, mais de um), tabagismo (sim, não), medicação para dormir (sim, não) e uso de fitoterápicos (sim, não).

O questionário PSQI-BR, versão traduzida e validada para o português do Brasil, foi utilizado para mensurar a qualidade do sono^(10)^. Esse questionário possui 19 questões organizadas em sete componentes: qualidade subjetiva do sono (C1), latência do sono (C2), duração do sono (C3), eficiência habitual do sono (C4), distúrbios do sono (C5), uso de medicação para dormir (C6) e disfunção diurna (C7), e cinco direcionadas ao cônjuge ou acompanhante de quarto, relativas aos hábitos de sono durante o último mês. Para este estudo não foram investigadas as questões relacionadas aos cônjuges. A soma das pontuações de cada item do instrumento resulta em um escore global que varia de 0 a 21. Um escore total maior que 5 indica que o indivíduo está apresentando grandes disfunções em pelo menos dois componentes, ou disfunção moderada em pelo menos três componentes^(10)^. Para este estudo optou-se por categorizar a variável qualidade do sono em “boa” (≤ 5) e “ruim” (> 5 pontos), conforme identificado previamente em estudo^(11)^.

A dosagem de cortisol foi realizada a partir de fios de cabelo ≥ 2 cm de comprimento (medidos com uso de régua metálica com o corte de 10 cm/0,1 mm), removidos o mais próximo possível da raiz, no vértice posterior da cabeça, e armazenados individualmente em recipientes, sem congelamento e protegidos da luz, conforme recomendação^(12)^.

As amostras foram submetidas à extração do cortisol e quantificação pelo método Enzyme-Linked Immunosorbent Assay – ELISA. Após a coleta, os fios foram lavados com isopropanol, seccionados, incubados em metanol (MeOH) a 50° C por 16 horas para extração de esteroides. Após secagem, o resíduo foi reconstituído em 250 µL de solução salina tamponada com fosfato (PBS) e submetido a amostra a vórtex para quantificação de cortisol, conforme protocolo adaptado^(13)^.

Considerando que o cabelo cresce em um ritmo relativamente constante de aproximadamente um centímetro por mês, afirma-se que os três centímetros de cabelo mais próximos ao couro cabeludo refletem os níveis de cortisol aos quais o indivíduo esteve exposto nos últimos três meses^(14)^. Para indivíduos saudáveis com baixos níveis de estresse, a mediana de concentração de cortisol capilar é de 55 picogramas de cortisol/miligramas de cabelos (pg/mg) (40–128 pg/mg) e de 250 pg/mg de cabelo (182–520 pg/mg) em indivíduos estressados^(14)^. Neste estudo, a média de cortisol capilar antes da intervenção foi de 600,30 pg/mg (DP = 6,03); assim, utilizou-se como ponto de corte menor ou igual a 600 pg/mg e maior que 600 pg/mg para as análises.

A abordagem individual incluiu informações sobre a pesquisa e critérios para participar. Para os interessados, aplicaram-se os critérios de elegibilidade e procedeu-se à entrega do Termo de Consentimento Livre e Esclarecido, tendo sido solicitada leitura e anuência de participação por meio da assinatura, uma via ficando com o participante e a outra retida pela equipe de pesquisa. Posteriormente, realizou-se a coleta de fios de cabelo, que foram armazenados em recipiente hermético, protegido da luz solar; entregues os questionários, sendo agendada uma data para devolução; e disponibilizadas as cápsulas de alecrim para a intervenção. Os participantes autorizaram o envio de mensagens via aplicativo de mensagens eletrônicas.

A intervenção consistiu em fornecer a cada participante um frasco com 60 cápsulas de 500 mg de extrato seco de *Rosmarinus officinalis L.* (alecrim) para ingerir com água, duas vezes ao dia (uma cápsula pela manhã, se possível, 30 minutos depois do café da manhã, e outra 12 horas após a primeira), totalizando 1 g/dia, por um período de 56 dias. O extrato seco P. S. 0,03% de Rosmarinus officinalis L. é originário das folhas da planta, com aspecto de um pó fino higroscópico sem presença de materiais estranhos, de cor bege esverdeado, com odor e sabor característicos, com cinzas totais de 63 em valores inferiores a 5,0%, densidade entre 0,300 e 0,600, pH entre 4,0 e 8,0, parcialmente solúvel em água, com umidade inferior a 10,0%, teor ativo de flavonoides maior que 0,03, contagem de bactérias aeróbias inferior a 10.000, contagem de fungos ou leveduras menor que 1.000 e de enterobactérias inferior a 1.000.

Para maior segurança e controle quanto à adesão ao tratamento proposto, foi disponibilizado, a cada participante, um minidiário para registro do horário das doses autoadministradas, das esquecidas, daquelas tomadas com atraso; e um folheto com orientações sobre a continuidade na ingestão de medicamentos de rotina, inclusive chás e fitoterápicos, as doses e horários de autoadministração do fitoterápico, o que fazer diante do esquecimento da dose, cuidados para a conservação e forma de contato com pesquisadores em caso dúvidas ou intercorrências. O participante foi orientado de que, nas situações de esquecimento de ingestão da dose diária, a cápsula esquecida deveria ser devolvida no término do período de intervenção (ao final dos 56 dias) ao pesquisador, como forma de controle das doses ingeridas.

Durante o período de intervenção, a cada cinco dias, eram enviadas mensagens via aplicativo para estimular o uso do fitoterápico e minimizar dúvidas. O participante foi orientado a comunicar imediatamente à equipe de pesquisa sobre alguma intercorrência decorrente do uso do fitoterápico, e decidir sobre permanecer no estudo ou desistir. Ao final dos 56 dias, foi agendada nova data de coleta de dados e de amostras de cabelo.

### Análise e Tratamento dos Dados

Os dados coletados formaram um banco no programa Microsoft Excel® a partir de dupla digitação independente, e foram analisados com auxílio do *software* SPSS, versão 21.0. Estão armazenados no computador pessoal da coordenadora da pesquisa, e poderão ser disponibilizados mediante consulta. As variáveis quantitativas foram expressas como média e desvio padrão na presença de distribuição normal, ou mediana e intervalo interquartil na presença de distribuição assimétrica. As variáveis categóricas foram apresentadas por meio de valores absolutos e relativos; as quantitativas, pela média e desvio padrão. Para investigar associação entre as variáveis categóricas foi utilizado o teste Qui-Quadrado de Pearson ou o teste Exato de Fisher. E para análise de amostras pareadas se utilizou o Teste de McNemar. Realizou-se análise de correlação de Spearmann para avaliar as relações entre as variáveis do estudo. Foram considerados intervalo de confiança de 95% e p < 0,05.

### Aspectos Éticos

O estudo está registrado no Registro Brasileiro de Ensaios Clínicos – Rebec (nº RBR 88hrnnw). Foi aprovado pelo Comitê de Ética em Pesquisa sob o n. 5.197.916, atendendo aos preceitos éticos de pesquisa conforme a Lei N. 14.874/2024, que dispõe sobre a pesquisa com seres humanos. A participação foi voluntária, e o consentimento para com a pesquisa ocorreu após a leitura e com a assinatura do Termo de Consentimento Livre e Esclarecido.

## RESULTADOS

Sobre os participantes, assinala-se que 41 recusaram a participação. Quando aplicados os critérios de elegibilidade, 11 foram excluídos (três estavam amamentando, uma era gestante, uma estava em tratamento para engravidar, dois possuíam cabelo de tamanho < 2 cm, um estava realizando tratamento no couro cabeludo, um apresentava doença digestiva e dois estavam em tratamento para diabetes mellitus). Após o início da intervenção, 12 profissionais interromperam o uso do fitoterápico e não participaram da última fase de coleta de dados. Ainda, houve um participante excluído por preenchimento incompleto do questionário do sono, o que inviabilizou a análise.

Participaram 35 trabalhadores de enfermagem com idade média de 39,67 anos (± 7,58). O percentual de 85,7% (n = 30) representa as mulheres, 80% (n = 28) tinham filhos, 74,3% (n = 26) possuíam companheiro. A maioria atuava na UPA (77,1%, n = 27) e eram técnicos ou auxiliares de enfermagem (77,1%, n = 27), com carga horária ≤ 44 h/semanais e possuíam um vínculo empregatício. Quanto ao uso de medicamentos, houve prevalência de profissionais que não utilizavam medicações para dormir (85,7%, n = 30) e que não utilizavam fitoterápico (97,1%, n = 34).

Verificou-se que, **antes da intervenção**, predominou a qualidade do sono ruim naqueles com companheiro (68,2%), com filhos (72,7%), com um vínculo de emprego e carga horária maior ou igual a 44 h/semana (77,3%). **Após a intervenção**, predominou a má qualidade do sono naqueles com companheiro e com filhos (66,7%), com um vínculo empregatício e carga horária menor ou igual a 44 h/ semana (86,7%).

Em relação aos níveis de cortisol, **antes da intervenção** observou-se que 86,7% (n = 13) das participantes do sexo de nascimento feminino apresentaram valores de cortisol maiores que 600 pg/mg, do mesmo modo aqueles que tinham companheiro (86,7%, n = 13) e que possuíam filhos (93,3%, n = 14). Apresentaram níveis de cortisol < 600 pg/mg a maioria dos profissionais que não faziam uso de medicação para dormir (90%, n = 18) e aqueles que não utilizavam fitoterápico (100%, n = 20).

Identificou-se que, **após a intervenção**, não houve associação significativa dos níveis de cortisol e as variáveis sociodemográficas e ocupacionais. Ou seja, nesta amostra, número de empregos e carga horária não afetaram os níveis de cortisol após a intervenção.

Na Tabela 1 constam dados relacionados à qualidade do sono e nível de cortisol antes e após a intervenção com alecrim.

**Tabela 1:** Relação da qualidade do sono antes e após a intervenção em profissionais de enfermagem de serviços de emergência – Ijuí, RS, Brasil, 2023. (n = 35).

	Qualidade do sono		p
	Boa	Ruim	
Antes	13 (37,1)	22 (62,9)	0,039*
Depois	20 (57,1)	15 (42,9)	
	**Cortisol capilar**		**p**
	**<600 pg/mg**	**≥600 pg/mg**	
Antes	20 (57,1)	15 (42,9)	<0,001*
Depois	2 (5,7)	33 (94,3)	

Fonte: Autoras, 2023.

*Teste McNemar.

Houve diferença significativa entre a qualidade do sono classificada como boa, ao se comparar o período antes com o após a intervenção (p = 0,039). Também se observou aumento da proporção de profissionais com níveis de cortisol maiores ou iguais a 600 pg/mg, na comparação entre o antes e depois da intervenção (p < 0,001).

Evidenciou-se que aqueles que utilizavam medicação para dormir apresentaram os piores valores de cortisol (p = 0,017). Não foi encontrada associação do uso de medicamento e fitoterápico com a qualidade do sono.

A Tabela 2 apresenta a matriz de correlação, na qual identifica-se que a qualidade do sono antes da intervenção se relacionou positivamente à qualidade do sono após a intervenção; nível de cortisol antes da intervenção relacionou-se positivamente com o número de vínculos de trabalho; e carga horária com o nível de cortisol após a intervenção.

**Tabela 2:** Matriz de correlação entre as variáveis de estudo de profissionais de enfermagem nos serviços de emergência – Ijuí, RS, Brasil, 2023. (n = 35).

	Idade	Vínculo de trabalho	CH	QS antes da intervenção	QS depois da intervenção	Cortisol antes da intervenção
Qualidade do sono antes	,054	−,057	−,044			
	,758	,746	,803			
Qualidade do sono depois	−,094	−,229	−,045	,673**		
	,590	,187	,799	**,000**		
Cortisol antes da intervenção	,205	,364*	,094	,058	−,277	
	,238	**,031**	,593	,739	,107	
Cortisol depois da intervenção	,035	,321	,360*	,000	−,190	,209
	,843	,060	**,034**	,998	,275	,228

Nota: CH – Carga horária; QS – Qualidade do Sono.

*Correlação significativa a nível de p ≤ 0,05.

**Correlação significativa a nível de p ≤ 0,01.

## DISCUSSÃO

Os resultados evidenciaram que a intervenção com 1 g de extrato seco de *Rosmarinus officinalis L.* (alecrim) melhorou a qualidade do sono de profissionais de enfermagem dos serviços de emergência, pois foi identificada diferença significativa na qualidade do sono antes e após intervenção; no entanto, não foi suficiente para reduzir os níveis de cortisol capilar no grupo investigado.

Neste estudo, predominou o sexo feminino atribuído no nascimento, resultado semelhante a estudo nacional realizado em unidades de urgência/emergência^(15)^. Estudo identificou que as mulheres apresentaram probabilidade de ter sono de pior qualidade cerca de duas vezes maior que os homens, o que pode estar relacionado a condições psicológicas, uma vez que apresentam sintomas de ansiedade e depressão com maior frequência que os homens e modificações hormonais, presentes durante ciclos menstruais, na gestação e menopausa^(1)^. Ainda, há o risco de estresse dada a natureza das condições de trabalho^(16)^, o que tem influência no padrão do sono.

O sono insatisfatório, resultante da privação do sono, tem efeitos sobre o humor que são comparáveis aos efeitos sobre o funcionamento cognitivo. Esta descoberta é relevante para os trabalhadores do serviço de urgência/emergência, dado os devido aos eventos críticos que envolvem o cuidado. Neste estudo, identificou-se predomínio da má qualidade do sono antes da intervenção (62,9%, n = 22), dado semelhante a estudos realizados com a enfermagem que utilizaram o mesmo instrumento de avaliação^(11)^.

A má qualidade do sono ocorre quando o indivíduo não consegue adormecer, acorda várias vezes durante a noite e não se sente descansado ao acordar, e esta condição está intimamente ligada à piora dos quadros de doenças cardiovasculares^(17)^. Evidências de uma metanálise levaram a concluir que os enfermeiros são os profissionais mais suscetíveis à má qualidade do sono^(18)^.

Outras variáveis podem influenciar a qualidade do sono, como ter filhos, carga horária e situação conjugal. Os resultados evidenciaram que antes e após a intervenção houve má qualidade do sono naqueles que possuíam filhos, o que coaduna parcialmente com os resultados de estudo que evidenciou que ter filhos pequenos influencia de maneira significativa a qualidade do sono dos pais^(19)^. A ausência de questionamento sobre a idade dos filhos impediu uma análise mais aprofundada dos dados.

Houve prevalência de má qualidade do sono naqueles com companheiro, dado divergente de pesquisa realizada com trabalhadores australianos que identificou que indivíduos casados apresentaram melhor qualidade do sono^(19)^.

O uso do alecrim melhorou a qualidade do sono. Antes da intervenção, 37,1% dos participantes apresentavam boa qualidade do sono; após a intervenção, esse percentual elevou-se para 57,1%. Da mesma forma, 62,9% dos participantes apresentavam qualidade do sono ruim; após a intervenção, esse percentual foi de 42,9%. Isso significa que a intervenção influenciou para a melhora da qualidade do sono nessa amostra. Ter boa qualidade do sono é importante para a manutenção da saúde, pois alguns mecanismos de depuração de toxinas ocorrem durante esse período e dada a sua relevância para as funções imunológicas, cardiovasculares, reprodutivas, endócrinas e para o controle da dor^(2)^.

O resultado do efeito do alecrim sobre a qualidade do sono é inovador em publicações brasileiras, e assemelha-se a resultados de pesquisa japonesa que identificou redução da sonolência diurna com ingestão de 1 g/dia de alimento teste contendo extrato seco de Rosmarinus officinalis L. por 4 semanas^(7)^. Estudo realizado com o alecrim na versão óleo essencial foi capaz de melhorar a qualidade do sono em indivíduos ansiosos e com dificuldades para relaxar, auxiliando na regulação do ciclo sono-vigília^(6)^.

Os benefícios do alecrim têm sido evidenciados na literatura, como na redução de sintomas de transtornos mentais comuns, como irritação, ansiedade, dificuldade para dormir e dificuldade de concentração, sem efeitos adversos^(20)^ e como auxiliar da dieta alimentar, com redução do índice de massa corporal e da relação cintura-quadril^(21)^.

Em relação ao **cortisol**, antes e após a intervenção houve prevalência no sexo feminino com valores > 600 pg/mg, o que caracteriza taxas elevadas, embora sem significância estatística. Pesquisa realizada com profissionais de enfermagem identificou elevados níveis de cortisol capilar, o que sugere a presença de estresse, porém sem significância estatística^(22)^. Outra pesquisa identificou associação entre níveis elevados de cortisol capilar e o estresse percebido^(4)^. Estudo destaca que intervenções focadas no bem-estar dos trabalhadores podem mitigar alguns desses efeitos negativos ao reduzirem o estresse e a ansiedade entre os enfermeiros^(23)^.

Pôde-se identificar aumento significativo dos níveis de cortisol capilar na amostra estudada após a intervenção, o que sugere estresse crônico neste grupo profissional. Isso pode estar relacionado a inconstâncias no processo de trabalho no setor de urgência/emergência, onde lidar com o constante estado de prontidão e sofrimento humano pode gerar impactos negativos à saúde e ao bem-estar dos profissionais de enfermagem. Indivíduos com níveis elevados de cortisol apresentaram aumentos significativos nos níveis de colesterol total, LDL e triglicerídeos^(24)^, estresse percebido^(4)^ e maior gravidade de insônia, depressão e ansiedade^(25)^.

Os dados também mostram correlação entre ter mais de um vínculo de trabalho e piores níveis de cortisol, assim como níveis maiores de cortisol naqueles que cumpriam carga horária mais elevada, evidenciando que quanto maiores o número de empregos e a carga horária, maior o nível de estresse. Os enfermeiros de emergência são altamente suscetíveis ao esgotamento devido à exposição contínua a eventos traumáticos, horários de trabalho variados, violência dirigida ao pessoal e, nos últimos tempos, devido aos fatores estressores decorrentes da pandemia de COVID-19^(26)^. O estresse daqueles que trabalham na emergência está relacionado ao aumento no cortisol capilar e a reações fisiológicas como dores lombares, fadiga, exaustão, rigidez no pescoço e acidez estomacal^(22)^.

Houve associação significativa entre cortisol e o uso de medicação para dormir após a intervenção, ou seja, os trabalhadores que utilizavam medicação para dormir apresentaram maiores níveis de cortisol. Estudo realizado com profissionais de enfermagem durante a pandemia de COVID-19 identificou maior prevalência de sono ruim relatado por 85,7% da amostra, e uso de medicamentos para dormir por 25,9% deles^(27)^, sugerindo que esses profissionais buscam soluções medicamentosas para melhorar a qualidade do sono.

Ao analisar a relação entre cortisol capilar e qualidade do sono, não foi identificada associação significativa antes e após a intervenção. Apesar disso, pesquisadores reforçam a importância de mensurar o cortisol capilar em profissionais de enfermagem, obtido nos cabelos, pois é um biomarcador confiável para mensurar o estresse^(16)^.

A má qualidade do sono relaciona-se a impactos negativos à saúde, como adoecimento físico^(12)^, transtornos do apetite, sensação de má digestão, flatulência, ganho de peso, irritabilidade, dor de cabeça, sensação de baixa autoestima, labilidade do humor^(2)^. Indivíduos que se encontram com privação de sono têm seus níveis de cortisol alterados como uma resposta ao estresse, pois a privação e a interrupção do sono afetam o ritmo circadiano e o sistema imunológico^(28)^.

Ao interpretar os resultados deste estudo, é importante salientar as limitações, como o pequeno tamanho da amostra e o uso de questionários autoadministrados, cujos dados podem ser tendenciosos. Outro ponto a ser considerado é a amostra do cabelo de 2 cm necessária para análise de cortisol que limitou o número de participantes do sexo masculino.

A ausência de efeito do alecrim no estresse crônico pode ser explicada pelo curto período de coleta de dados. Pesquisadores sugerem que o cortisol capilar reflete variações hormonais em escalas de tempo mais longas, como meses a um ano, e não é sensível a mudanças rápidas associadas ao estresse agudo^(29)^. Assim, são necessários estudos adicionais, com delineamentos longitudinais, que considerem um período maior de acompanhamento e com amostras maiores que poderão ter o potencial de ampliar a compreensão dos efeitos do alecrim no cortisol na perspectiva de desenvolver intervenções preventivas e de tratamento mais eficazes. Pesquisas futuras podem desvendar os efeitos duradouros do alecrim na saúde humana.

O estudo contribui com dados referentes ao trabalho de enfermagem em serviços de urgência/emergência e pode contribuir no planejamento de ações que promovam a saúde do trabalhador por meio de práticas integrativas em saúde. Ainda, fornece dados sobre a avaliação do cortisol capilar, ampliando as possibilidades de investigação deste biomarcador. Os resultados fornecem evidências para o possível efeito do alecrim na qualidade do sono, com possibilidades de desenvolvimento de novas estratégias preventivas e de tratamento.

## CONCLUSÃO

A intervenção com 1 g de extrato seco de alecrim melhorou significativamente a qualidade do sono, e foi associada ao aumento dos níveis de cortisol de profissionais de enfermagem nos serviços de urgência/emergência. As evidências limitadas sobre o tema requerem aprofundamento, e os dados sugerem o desenvolvimento de estratégias que contribuam para minimizar o estresse e melhorar a qualidade do sono desses profissionais.

## DISPONIBILIDADE DE DADOS

Todo o conjunto de dados que dá suporte aos resultados deste estudo está disponível mediante solicitação ao autor correspondente Nathália Fortes Schlotfeldt. O conjunto de dados não está publicamente disponível devido ainda não ter o prazo mínimo de dois anos, requisito estabelecido para sua disponibilização.

# Translation: Análisis del uso de romero para los niveles de cortisol y la calidad del sueño en profesionales de enfermería

**DOI:** 10.1590/1980-220X-REEUSP-2025-0050es

**Authors:** Nathália Fortes Schlotfeldt, Rosângela Marion da Silva, Etiane de Oliveira Freitas, Fernanda Moura D’Almeida Miranda, Carolina Renz Pretto, Flávia Camef Dorneles Lenz

**Affiliations:** 1 Universidade Federal de Santa Maria, Programa de Pós-Graduação em Enfermagem, Santa Maria, RS, Brasil.; 2 Universidade Federal do Paraná, Curitiba, PR, Brasil.; 3 Secretaria Municipal de Saúde de Ijuí, Serviço de Atendimento Móvel de Urgência, Ijuí, RS, Brasil.

**Keywords:** Enfermería de Urgencia, Sueño, Rosmarinus, Hidrocortisona

## Abstract

**Objetivo:**

Analizar la eficacia de 1 g de extracto seco de romero en la calidad del sueño y el cortisol capilar de profesionales de enfermería de urgencias/emergencias.

**Método:**

Estudio de intervención, cuasi experimental, de tipo antes y después, realizado entre septiembre y noviembre de 2022 con profesionales de enfermería de un servicio de urgencias hospitalario y de un servicio de urgencias de Rio Grande do Sul. Se utilizaron un cuestionario sociodemográfico/ocupacional, el Índice de Calidad del Sueño de Pittsburgh y muestras de cabello. La intervención consistió en la ingesta de cápsulas de extracto seco de romero de 500 mg, dos veces al día, durante 56 días. Análisis descriptivo y analítico, con pruebas de asociación y correlación (p < 0,05).

**Resultados:**

Participaron 35 profesionales. Predominaron la mala calidad del sueño y los niveles elevados de cortisol capilar. La intervención mejoró significativamente el sueño (p = 0,039), sin reducción de los niveles de cortisol.

**Conclusión:**

La intervención con romero mejoró la calidad del sueño, pero no fue suficiente para reducir las concentraciones de cortisol capilar, por lo que se necesitan otras estrategias que contribuyan a la salud de los trabajadores.

## INTRODUCCIÓN

El sueño es importante para la homeostasis, momento en el que se interrumpe el estado de vigilia, lo que conduce a una disminución de las actividades metabólicas, la relajación muscular y la reducción de las actividades sensoriales^(1)^. El sueño interfiere en las funciones cognitivas, la consolidación de la memoria y el almacenamiento de información, y es esencial para el equilibrio del organismo humano^(2)^.

Los trastornos del sueño afectan a la capacidad de dormir y se han asociado a alteraciones en los niveles de cortisol, conocida como la hormona del estrés, con impacto en la salud física y mental. El cortisol sigue un ciclo circadiano natural, alcanza su pico por la mañana para preparar el cuerpo para las actividades diarias y disminuye a lo largo del día para facilitar el sueño. Sin embargo, los problemas de sueño, como el insomnio o la mala calidad del sueño, pueden alterar este ritmo al mantener niveles elevados de cortisol por la noche, lo que contribuye al estrés crónico y la fatiga^(3)^.

Esta situación, sumada a las exigencias del trabajo y al trabajo por turnos, sugiere el deterioro de la salud del trabajador. Los profesionales que trabajan por turnos, como los enfermeros que prestan servicios de urgencia y emergencia, se enfrentan a situaciones de estrés, presión de tiempo y exigencias físicas^(4)^. El trabajo en turnos nocturnos, en particular, implica una desincronización circadiana, ya que el trabajador debe permanecer despierto durante el período en que el organismo, fisiológicamente, está preparado para el descanso, lo que alerta sobre la importancia de intervenciones que puedan contribuir a la calidad del sueño y la salud del trabajador.

Para mejorar la calidad del sueño, se cita como alternativa el uso de plantas medicinales, como el Rosmarinus officinalis L. (romero). Esta planta se utiliza ampliamente como conservante natural de alimentos y presenta acciones farmacológicas que incluyen antibacterianas, antidiabéticas, antiinflamatorias, antitumorales y antioxidantes^(5)^. A pesar de las limitadas investigaciones de intervención con el romero, las evidencias apuntan a beneficios como la reducción de la ansiedad, la mejora de la depresión y de la calidad del sueño^(6)^, y la disminución de la somnolencia diurna^(7)^. Estos resultados sugieren que el romero es una posibilidad terapéutica para mejorar el sueño; sin embargo, no hay evidencias que relacionen su uso con la mejora del estrés.

El estrés crónico puede evaluarse mediante la medición del cortisol capilar. Las muestras de cabello tienen la ventaja de detectar el cortisol a lo largo de varios meses y suponen un procedimiento no invasivo, además de ser fáciles de almacenar y resistentes a la descomposición, a diferencia de los fluidos corporales^(8)^.

Así, teniendo en cuenta la escasa bibliografía disponible sobre los efectos del romero en la salud humana, el reducido número de estudios de intervención recientes sobre el cortisol capilar y que los profesionales de enfermería son susceptibles al estrés, se identifica la importancia de analizar la eficacia de 1 g de extracto seco de romero en la calidad del sueño y la concentración de cortisol capilar de profesionales de enfermería de servicios de urgencia/emergencia, objetivo de esta investigación.

## MÉTODO

### Tipo de Estudio

Se trata de un estudio cuasi experimental, con un solo grupo, pre y post intervención.

### Lugar y Población

Se llevó a cabo en un municipio de la región noroeste del estado de Rio Grande do Sul, en una Unidad de Atención de Urgencias (UPA) 24 horas, institución pública que presta asistencia de urgencia y emergencia a nivel intermedio a una población de 83 764 habitantes y cuenta con 43 profesionales de enfermería; y un servicio de urgencias hospitalario (SUH), que forma parte de una institución filantrópica de referencia en la atención en salud de toda la región cubierta por la 17ª Coordinación Regional de Salud, que abarca una población de aproximadamente 231 229 personas y cuenta con 57 profesionales de enfermería en el sector.

### Criterios de Selección

Se incluyeron los técnicos y auxiliares de enfermería y los enfermeros que llevaban al menos un mes trabajando en los servicios de emergencia de las instituciones seleccionadas, independientemente de su tipo de contrato. La delimitación del tiempo de servicio tuvo en cuenta el Índice de Calidad del Sueño de Pittsburgh, que evalúa la calidad del sueño durante el mes anterior.

En cuanto a los criterios de exclusión, se descartaron: profesionales que presentaban hipersensibilidad al romero; mujeres embarazadas, lactantes, epilépticos, personas en tratamiento por dislipidemia o diabetes mellitus, profesionales con disfunción biliar o hepática, con enfermedades prostáticas o gastroenteritis, debido a la falta de estudios que garanticen la seguridad de su uso en estas condiciones; profesionales con cabellos < 2 cm en la región del vórtice posterior de la cabeza o que estaban utilizando medicación tópica en el cuero cabelludo durante el periodo de recolección de datos, para prevenir interferencias en la concentración de cortisol; o ausentes del trabajo por cualquier motivo durante el periodo de recolección de datos.

### Definición de la Muestra

El muestreo no fue aleatorio y la muestra mínima se estableció mediante un cálculo muestral, aplicando una expresión para proporciones pareadas^(9)^, en la que se arbitró una proporción del 50% de concordantes, el 10% de discordantes, una certeza (potencia de la prueba) del 80% y un nivel de significación del 5%. Estos parámetros dieron como resultado una muestra mínima de 19 participantes, teniendo en cuenta que cada uno sería su propio par en la evaluación de los resultados antes y después de la intervención. La población del estudio estuvo compuesta por 100 profesionales de enfermería, todos los cuales fueron invitados a participar en la investigación.

### Recolección de Datos

La realización de la investigación comprendió dos etapas de recolección de datos (antes y después de la intervención) y un periodo de intervención. La recolección se llevó a cabo de septiembre a noviembre de 2022, de forma individual y presencial, en el lugar de trabajo de los participantes, con cita previa y según disponibilidad.

En la recolección colaboraron de forma simultánea dos personas, una estudiante de enfermería, becaria de iniciación científica, que recibió capacitación y formación para la recolección de datos, y una enfermera con experiencia en recolección de datos cuantitativos, doctoranda en enfermería.

### Instrumento de Recolección de Datos

Los instrumentos de recolección de datos comprendieron cuestionarios autoaplicables, con variables sociodemográficas, y el Índice de Calidad del Sueño de Pittsburgh (PSQI-BR). También se recolectaron muestras de cabello para análisis de cortisol capilar.

El cuestionario sociodemográfico y clínico se utilizó para caracterizar a los participantes e incluyó la investigación de las siguientes variables: edad (en años), sexo asignado al nacer (masculino, femenino), situación conyugal (con pareja/sin pareja), hijos (sí, no), lugar de trabajo (UPA, PSH), cargo (enfermero, técnico de enfermería, auxiliar de enfermería), carga horaria (≤ 44 h/semana, > 44 h/semana), número de vínculos laborales (uno, más de uno), tabaquismo (sí, no), medicación para dormir (sí, no) y uso de fitoterápicos (sí, no).

El cuestionario PSQI-BR, traducido y validado al portugués de Brasil, se utilizó para medir la calidad del sueño^(10)^. Este cuestionario consta de 19 preguntas organizadas en siete componentes: calidad subjetiva del sueño (C1), latencia del sueño (C2), duración del sueño (C3), eficiencia habitual del sueño (C4), trastornos del sueño (C5), uso de medicación para dormir (C6) y disfunción diurna (C7), y cinco dirigidas al cónyuge o acompañante de habitación, relativas a los hábitos de sueño durante el último mes. Para este estudio no se investigaron las preguntas relacionadas con los cónyuges. La suma de las puntuaciones de cada ítem del instrumento da como resultado una puntuación global que varía de 0 a 21. Una puntuación total superior a 5 indica que el individuo presenta disfunciones graves en al menos dos componentes, o disfunción moderada en al menos tres componentes^(10,11)^.

La medición del cortisol se realizó a partir de cabellos de ≥ 2 cm de longitud (medidos con una regla metálica con corte de 10 cm/0,1 mm), extraídos lo más cerca posible de la raíz, en el vértice posterior de la cabeza, y almacenados individualmente en recipientes, sin congelar y protegidos de la luz, según las recomendaciones^(12)^.

Las muestras se sometieron a extracción de cortisol y cuantificación mediante el método Enzyme-Linked Immunosorbent Assay – ELISA. Tras la recolección, los cabellos se lavaron con isopropanol, se seccionaron y se incubaron en metanol (MeOH) a 50 °C durante 16 horas para la extracción de esteroides. Tras el secado, el residuo se reconstituyó en 250 µL de solución salina tamponada con fosfato (PBS) y se sometió a vórtex para la cuantificación del cortisol, según el protocolo adaptado^(13)^.

Teniendo en cuenta que el cabello crece a un ritmo relativamente constante de aproximadamente un centímetro al mes, se afirma que los tres centímetros de cabello más cercanos al cuero cabelludo reflejan los niveles de cortisol a los que ha estado expuesto el individuo en los últimos tres meses^(14)^. Para individuos sanos con bajos niveles de estrés, la mediana de la concentración de cortisol capilar es de 55 picogramos de cortisol/miligramos de cabello (pg/mg) (40–128 pg/mg) y de 250 pg/mg de cabello (182–520 pg/mg) en individuos estresados^(14)^. En este estudio, la media de cortisol capilar antes de la intervención fue de 600,30 pg/mg (DE = 6,03); por lo tanto, se utilizó como punto de corte menor o igual a 600 pg/mg y mayor que 600 pg/mg para los análisis.

El enfoque individual incluyó información sobre la investigación y los criterios para participar. A los interesados se les aplicaron los criterios de elegibilidad y se les entregó el Término de Consentimiento Libre y Esclarecido, que debían leer y firmar para dar su consentimiento a participar, quedando una copia con el participante y otra con el equipo de investigación. Posteriormente, se llevó a cabo la recolección de hebras de cabello, que se almacenaron en un recipiente hermético, protegido de la luz solar; se entregaron los cuestionarios, se programó una fecha para su devolución y se proporcionaron las cápsulas de romero para la intervención. Los participantes autorizaron el envío de mensajes a través de una aplicación de mensajería electrónica.

La intervención consistió en proporcionar a cada participante un frasco con 60 cápsulas de 500 mg de extracto seco de *Rosmarinus officinalis L.* (romero) para ingerir con agua, dos veces al día (una cápsula por la mañana, si es posible, 30 minutos después del desayuno, y otra 12 horas después de la primera), con un total de 1 g/día, durante un periodo de 56 días. El extracto seco P. S. 0,03% de Rosmarinus officinalis L. procede de las hojas de la planta, con aspecto de polvo fino higroscópico sin presencia de materiales extraños, de color beige verdoso, con olor y sabor característicos, con un contenido total de cenizas inferior al 5,0%, densidad entre 0,300 y 0,600, pH entre 4,0 y 8,0, parcialmente soluble en agua, con humedad inferior al 10,0%, contenido activo de flavonoides superior al 0,03, recuento de bacterias aeróbicas inferior a 10 000, recuento de hongos o levaduras inferior a 1000 y de enterobacterias inferior a 1000.

Para mayor seguridad y control en cuanto al cumplimiento del tratamiento propuesto, se proporcionó a cada participante un mini-diario para registrar la hora de las dosis autoadministradas, las olvidadas y las tomadas con retraso; y un folleto con instrucciones sobre la continuidad en la ingesta de medicamentos de rutina, incluidos tés y fitoterápicos, las dosis y horarios de autoadministración del fitoterápico, qué hacer en caso de olvido de la dosis, cuidados para la conservación y forma de contacto con los investigadores en caso de dudas o complicaciones. Se indicó al participante que, en caso de olvidar la dosis diaria, la cápsula olvidada debía devolverse al investigador al final del periodo de intervención (al cabo de 56 días), como forma de controlar las dosis ingeridas.

Durante el periodo de intervención, cada cinco días se enviaban mensajes a través de la aplicación para estimular el uso del fitoterápico y minimizar las dudas. Se indicó al participante que comunicara inmediatamente al equipo de investigación cualquier complicación derivada del uso del fitoterápico y que decidiera si deseaba continuar en el estudio o retirarse. Al final de los 56 días, se programó una nueva fecha para la recolección de datos y muestras de cabello.

### Análisis y Tratamiento de los Datos

Los datos recopilados se introdujeron en una base de datos en Microsoft Excel® mediante doble entrada independiente y se analizaron con ayuda del *software* SPSS, versión 21.0. Se almacenaron en la computadora personal de la coordinadora de la investigación y pueden consultarse previa solicitud. Las variables cuantitativas se expresaron como media y desviación estándar en presencia de distribución normal, o mediana e intervalo intercuartílico en presencia de distribución asimétrica. Las variables categóricas se presentaron mediante valores absolutos y relativos; las cuantitativas, mediante la media y la desviación estándar. Para investigar la asociación entre las variables categóricas se utilizó la prueba de chi cuadrado de Pearson o la prueba exacta de Fisher. Y para el análisis de muestras emparejadas se utilizó la prueba de McNemar. Se realizó un análisis de correlación de Spearmann para evaluar las relaciones entre las variables del estudio. Se consideraron un intervalo de confianza del 95% y p < 0,05.

### Aspectos Éticos

El estudio está registrado en el Registro Brasileño de Ensayos Clínicos (Rebec, n.º RBR 88hrnnw). Fue aprobado por el Comité de Ética en Investigación con el n.º 5.197.916, cumpliendo con los preceptos éticos de investigación según la Ley N.º 14.874/2024, que dispone sobre la investigación con seres humanos. La participación fue voluntaria y el consentimiento para la investigación se obtuvo tras la lectura y firma del Término de Consentimiento Libre y Esclarecido.

## RESULTADOS

En cuanto a los participantes, cabe señalar que 41 rechazaron participar. Al aplicar los criterios de elegibilidad, se excluyó a 11 (tres estaban amamantando, una estaba embarazada, una estaba en tratamiento para quedar embarazada, dos tenían el cabello de menos de 2 cm, uno estaba en tratamiento en el cuero cabelludo, uno presentaba una enfermedad digestiva y dos estaban en tratamiento para la diabetes mellitus). Tras el inicio de la intervención, 12 profesionales interrumpieron el uso del fitoterápico y no participaron en la última fase de recolección de datos. Además, se excluyó a un participante por no completar el cuestionario sobre el sueño, lo que imposibilitó el análisis.

Participaron 35 trabajadores de enfermería con una edad media de 39,67 años (± 7,58). El porcentaje de mujeres fue del 85,7% (n = 30), el 80% (n = 28) tenían hijos y el 74,3% (n = 26) tenían pareja. La mayoría trabajaba en la UPA (77,1%, n = 27) y eran técnicos o auxiliares de enfermería (77,1%, n = 27), con una carga horaria ≤ 44 h/semana y tenían un vínculo laboral. En cuanto al uso de medicamentos, hubo una prevalencia de profesionales que no utilizaban medicamentos para dormir (85.7%, n = 30) y que no utilizaban fitoterápicos (97.1%, n = 34).

Se observó que, **antes de la intervención**, predominaba la mala calidad del sueño en aquellos con pareja (68,2%), con hijos (72,7%), con un vínculo laboral y una carga horaria mayor o igual a 44 h/semana (77,3%). **Después de la intervención**, predominó la mala calidad del sueño en aquellas personas con pareja e hijos (66,7%), con un vínculo laboral y una carga horaria menor o igual a 44 h/semana (86,7%).

En cuanto a los niveles de cortisol, **antes de la intervención** se observó que el 86,7% (n = 13) de las participantes de sexo femenino presentaban valores de cortisol superiores a 600 pg/mg, al igual que aquellas que tenían pareja (86,7%, n = 13) e hijos (93,3%, n = 14). La mayoría de los profesionales que no tomaban medicación para dormir (90%, n = 18) y los que no utilizaban fitoterápicos (100%, n = 20) presentaban niveles de cortisol < 600 pg/mg.

Se identificó que, **después de la intervención**, no hubo una asociación significativa entre los niveles de cortisol y las variables sociodemográficas y ocupacionales. Es decir, en esta muestra, el número de empleos y la carga horaria no afectaron los niveles de cortisol después de la intervención.

En la Tabla 1 se presentan datos relacionados con la calidad del sueño y el nivel de cortisol antes y después de la intervención con romero.

**Tabla 1:** Relación de la calidad del sueño antes y después de la intervención en profesionales de enfermería de servicios de urgencia – Ijuí, RS, Brasil, 2023. (n = 35).

	Calidad del sueño		p
	Buena	Pobre	
Antes de	13 (37,1)	22 (62,9)	0,039*
Después	20 (57,1)	15 (42,9)	
	**Cortisol capilar**		**p**
	**<600 pg/mg**	**≥600 pg/mg**	
Antes de	20 (57,1)	15 (42,9)	<0,001*
Después de	2 (5,7)	33 (94,3)	

Fuente: Autoras, 2023.

*Prueba de McNemar.

Hubo una diferencia significativa entre la calidad del sueño clasificada como buena al comparar el periodo anterior y posterior a la intervención (p = 0,039). También hubo un aumento en la proporción de profesionales con niveles de cortisol mayores o iguales a 600 pg/mg al comparar antes y después de la intervención (p < 0,001).

Los que utilizaban medicación para dormir tenían los niveles de cortisol más bajos (p = 0,017). No se encontró ninguna asociación entre el uso de medicamentos y hierbas medicinales y la calidad del sueño.

La Tabla 2 muestra la matriz de correlaciones, que muestra que la calidad del sueño antes de la intervención estaba positivamente correlacionada con la calidad del sueño después de la intervención; el nivel de cortisol antes de la intervención estaba positivamente correlacionado con el número de trabajos; y la carga de trabajo con el nivel de cortisol después de la intervención.

**Tabla 2:** Matriz de correlación entre las variables de estudio de los profesionales de enfermería de los servicios de urgencias – Ijuí, RS, Brasil, 2023. (n = 35).

	Edad	Relación laboral	CH	CS antes de la intervención	CS después de la intervención	Cortisol antes de la intervención
Calidad del sueño antes	,054	−,057	−,044			
	,758	,746	,803			
Calidad del sueño después de	−,094	−,229	−,045	,673**		
	,590	,187	,799	**,000**		
Cortisol antes de la intervención	,205	,364*	,094	,058	−,277	
	,238	**,031**	,593	,739	,107	
Cortisol después de la intervención	,035	,321	,360*	,000	−,190	,209
	,843	,060	**,034**	,998	,275	,228

Nota: CH: carga horaria; CS: calidad del sueño.

*Correlación significativa a p ≤ 0,05.

**Correlación significativa a p ≤ 0,01.

## DISCUSIÓN

Los resultados evidenciaron que la intervención con 1 g de extracto seco de *Rosmarinus officinalis L.* (romero) mejoró la calidad del sueño de los profesionales de enfermería de los servicios de urgencias, ya que se identificó una diferencia significativa en la calidad del sueño antes y después de la intervención; sin embargo, no fue suficiente para reducir los niveles de cortisol capilar en el grupo investigado.

En este estudio predominó el sexo femenino asignado al nacer, un resultado similar al de un estudio nacional realizado en unidades de urgencia/emergencia^(15)^. El estudio identificó que las mujeres tenían aproximadamente el doble de probabilidades de tener un sueño de peor calidad que los hombres, lo que puede estar relacionado con condiciones psicológicas, ya que presentan síntomas de ansiedad y depresión con mayor frecuencia que los hombres, y con cambios hormonales, presentes durante los ciclos menstruales, el embarazo y la menopausia^(1)^. Además, existe el riesgo de estrés debido a la naturaleza de las condiciones de trabajo^(16)^, lo que influye en los patrones de sueño.

El sueño insatisfactorio, resultado de la privación del sueño, tiene efectos sobre el estado de ánimo que son comparables a los efectos sobre el funcionamiento cognitivo. Este hallazgo es relevante para los trabajadores de los servicios de urgencia/emergencia, dados los eventos críticos que implican los cuidados. En este estudio, se identificó un predominio de la mala calidad del sueño antes de la intervención (62,9%, n = 22), dato similar al de estudios realizados con personal de enfermería que utilizaron el mismo instrumento de evaluación^(11)^.

La mala calidad del sueño se produce cuando el individuo no puede conciliar el sueño, se despierta varias veces durante la noche y no se siente descansado al despertar, y esta condición está íntimamente relacionada con el empeoramiento de las enfermedades cardiovasculares^(17)^. Las pruebas de un metaanálisis llevaron a la conclusión de que los enfermeros son los profesionales más susceptibles a la mala calidad del sueño^(18)^.

Otras variables pueden influir en la calidad del sueño, como tener hijos, la carga horaria y la situación conyugal. Los resultados evidenciaron que, antes y después de la intervención, hubo mala calidad del sueño en aquellos que tenían hijos, lo que concuerda parcialmente con los resultados de un estudio que evidenció que tener hijos pequeños influye de manera significativa en la calidad del sueño de los padres^(19)^. La ausencia de preguntas sobre la edad de los hijos impidió un análisis más profundo de los datos.

Hubo una prevalencia de mala calidad del sueño en aquellos con pareja, un dato divergente de una investigación realizada con trabajadores australianos que identificó que los individuos casados presentaban una mejor calidad del sueño^(19)^.

El uso del romero mejoró la calidad del sueño. Antes de la intervención, el 37,1% de los participantes presentaba una buena calidad del sueño; después de la intervención, este porcentaje aumentó al 57,1%. Del mismo modo, el 62,9% de los participantes presentaba una mala calidad del sueño; después de la intervención, este porcentaje fue del 42,9%. Esto significa que la intervención influyó en la mejora de la calidad del sueño en esta muestra. Tener una buena calidad del sueño es importante para mantener la salud, ya que algunos mecanismos de depuración de toxinas se producen durante este periodo y dada su relevancia para las funciones inmunológicas, cardiovasculares, reproductivas, endocrinas y para el control del dolor^(2)^.

El resultado del efecto del romero sobre la calidad del sueño es innovador en las publicaciones brasileñas y se asemeja a los resultados de una investigación japonesa que identificó una reducción de la somnolencia diurna con la ingesta de 1 g/día de un alimento de prueba que contenía extracto seco de Rosmarinus officinalis L. durante 4 semanas^(7)^. Un estudio realizado con romero en forma de aceite esencial fue capaz de mejorar la calidad del sueño en personas ansiosas y con dificultades para relajarse, ayudando a regular el ciclo sueño-vigilia^(6)^.

Los beneficios del romero han sido demostrados en la literatura, como la reducción de los síntomas de trastornos mentales comunes, tales como irritabilidad, ansiedad, dificultad para dormir y dificultad para concentrarse, sin efectos adversos^(20)^, y como complemento de la dieta, con reducción del índice de masa corporal y de la relación cintura-cadera^(21)^.

En relación con el **cortisol**, antes y después de la intervención hubo prevalencia en el sexo femenino con valores > 600 pg/mg, lo que caracteriza tasas elevadas, aunque sin significación estadística. Una investigación realizada con profesionales de enfermería identificó niveles elevados de cortisol capilar, lo que sugiere la presencia de estrés, aunque sin significación estadística^(22)^. Otra investigación identificó una asociación entre los niveles elevados de cortisol capilar y el estrés percibido^(4)^. Un estudio destaca que las intervenciones centradas en el bienestar de los trabajadores pueden mitigar algunos de estos efectos negativos al reducir el estrés y la ansiedad entre los enfermeros^(23)^.

Se pudo identificar un aumento significativo de los niveles de cortisol capilar en la muestra estudiada después de la intervención, lo que sugiere estrés crónico en este grupo profesional. Esto puede estar relacionado con las inconstancias en el proceso de trabajo en el sector de urgencias/emergencias, donde lidiar con el estado constante de alerta y el sufrimiento humano puede generar impactos negativos en la salud y el bienestar de los profesionales de enfermería. Las personas con niveles elevados de cortisol presentaron aumentos significativos en los niveles de colesterol total, LDL y triglicéridos^(24)^, estrés percibido^(4)^ y mayor gravedad de insomnio, depresión y ansiedad^(25)^.

Los datos también muestran una correlación entre tener más de un vínculo laboral y peores niveles de cortisol, así como niveles más altos de cortisol en aquellos que cumplían con una carga horaria más elevada, lo que evidencia que cuanto mayor es el número de empleos y la carga horaria, mayor es el nivel de estrés. Los enfermeros de urgencias son muy susceptibles al agotamiento debido a la exposición continua a eventos traumáticos, horarios de trabajo variables, violencia dirigida al personal y, más recientemente, a los factores estresantes derivados de la pandemia de COVID-19^(26)^. El estrés de quienes trabajan en emergencias está relacionado con el aumento del cortisol capilar y reacciones fisiológicas como dolor lumbar, fatiga, agotamiento, rigidez en el cuello y acidez estomacal^(22)^.

Se observó una asociación significativa entre el cortisol y el uso de medicamentos para dormir después de la intervención, es decir, los trabajadores que utilizaban medicamentos para dormir presentaban niveles más altos de cortisol. Un estudio realizado con profesionales de enfermería durante la pandemia de COVID-19 identificó una mayor prevalencia de sueño deficiente reportado por el 85,7% de la muestra, y el uso de medicamentos para dormir por el 25,9% de ellos^(27)^, lo que sugiere que estos profesionales buscan soluciones farmacológicas para mejorar la calidad del sueño.

Al analizar la relación entre el cortisol capilar y la calidad del sueño, no se identificó una asociación significativa antes y después de la intervención. A pesar de ello, los investigadores refuerzan la importancia de medir el cortisol capilar en los profesionales de enfermería, obtenido en el cabello, ya que es un biomarcador fiable para medir el estrés^(16)^.

La mala calidad del sueño se relaciona con impactos negativos para la salud, como enfermedades físicas^(12)^, trastornos del apetito, sensación de mala digestión, flatulencia, aumento de peso, irritabilidad, dolor de cabeza, sensación de baja autoestima, inestabilidad del estado de ánimo^(2)^. Las personas que sufren privación del sueño presentan alteraciones en los niveles de cortisol como respuesta al estrés, ya que la privación y la interrupción del sueño afectan al ritmo circadiano y al sistema inmunológico^(28)^.

Al interpretar los resultados de este estudio, es importante destacar las limitaciones, como el pequeño tamaño de la muestra y el uso de cuestionarios autoadministrados, cuyos datos pueden ser sesgados. Otro punto a considerar es la muestra de cabello de 2 cm necesaria para el análisis de cortisol, lo que limitó el número de participantes masculinos.

La ausencia de efecto del romero en el estrés crónico puede explicarse por el corto periodo de recolección de datos. Los investigadores sugieren que el cortisol capilar refleja variaciones hormonales en escalas de tiempo más largas, como meses o un año, y no es sensible a los cambios rápidos asociados al estrés agudo^(29)^. Por lo tanto, se necesitan estudios adicionales, con diseños longitudinales, que consideren un período de seguimiento más largo y con muestras más grandes que puedan tener el potencial de ampliar la comprensión de los efectos del romero en el cortisol con miras a desarrollar intervenciones preventivas y tratamientos más eficaces. Investigaciones futuras pueden revelar los efectos duraderos del romero en la salud humana.

El estudio aporta datos relativos al trabajo de enfermería en servicios de urgencia/emergencia y puede contribuir a la planificación de acciones que promuevan la salud del trabajador mediante prácticas integradoras en salud. Además, proporciona datos sobre la evaluación del cortisol capilar, ampliando las posibilidades de investigación de este biomarcador. Los resultados proporcionan evidencia del posible efecto del romero en la calidad del sueño, con posibilidades de desarrollo de nuevas estrategias preventivas y de tratamiento.

## CONCLUSIÓN

La intervención con 1 g de extracto seco de romero mejoró significativamente la calidad del sueño y se asoció con el aumento de los niveles de cortisol de los profesionales de enfermería en los servicios de urgencia/emergencia. Las pruebas limitadas sobre el tema requieren una mayor investigación, y los datos sugieren el desarrollo de estrategias que contribuyan a minimizar el estrés y mejorar la calidad del sueño de estos profesionales.

## DISPONIBILIDAD DE DATOS

El conjunto completo de datos que respalda los hallazgos de este estudio está disponible previa solicitud a la autora correspondiente, Nathália Fortes Schlotfeldt. El conjunto de datos no está disponible públicamente porque aún no ha alcanzado el período mínimo de validez de dos años requerido para su disponibilidad.
